# A substance use relapse prevention programme for youth with substance use problems in Botswana

**DOI:** 10.4102/hsag.v30i0.2965

**Published:** 2025-07-30

**Authors:** Wada Gaolaolwe, Miriam M. Moagi, Gaotswake P. Kovane, Leepile A. Sehularo

**Affiliations:** 1 NuMIQ Research Focus Area, Faculty of Health Sciences, North-West University, Mahikeng, South Africa; 2 School of Nursing, Faculty of Health Sciences, University of Botswana, Gaborone, Botswana; 3 Department of Nursing, Faculty of Health Sciences, University of Limpopo, Limpopo, South Africa; 4 Lifestyle Diseases Research Focus Area, Faculty of Health Sciences, North-West University, Mahikeng, South Africa

**Keywords:** addiction, aftercare, substance use, programme, recovery, relapse, prevention, youth

## Abstract

**Background:**

Substance use relapse among the youth is a worldwide concern with negative ramifications on their lives, families, communities and nations at large.

**Aim:**

The purpose of this study was to develop and validate a programme for the prevention of substance use relapse among youth with substance use problems in Lobatse, Botswana.

**Setting:**

The research was conducted at a referral psychiatric hospital in Lobatse, Botswana.

**Methods:**

A multiphase approach was used, which included an integrative literature review in phase one and two qualitative studies (exploratory, descriptive and contextual design) in the empirical phase. The survey list of the practice-oriented theory was used for concept classification and guided the programme’s development.

**Results:**

The findings from the studies indicate that the programme for preventing substance use relapse among youth should focus more on aftercare initiatives such as tracking and assessment of those under this programme.

**Conclusion:**

A substance use relapse prevention programme for the youth should entail and provide impetus on the aftercare of the youth substance users to prevent their relapses.

**Contribution:**

The programme provides a robust strategy for preventing substance use relapse with actionable approaches for mental health professionals to effectively address the unique needs and circumstances of youth substance users through ongoing support and active engagement in recovery activities.

## Introduction

Substance use relapse (SUR) among the youth is a significant public health concern that endangers their lives and has a negative impact on their families and societies (Oladele 2021). Existing research highlights that young people worldwide are particularly vulnerable to substance use and related addiction because of influences such as peer pressure, accessibility of substances and media portrayals of substance use as pleasurable and glamorous that cumulatively contribute significantly to the initiation and persistence of substance use among youth (Feinstein, Richter & Foster 2012; Okafor 2020). Youth are the most affected demographic regarding substance use, which often begins during this critical period (Degenhardt et al. 2016). Approximately 65% to 85% of young people with substance use disorders (SUDs) relapse within a year of treatment (Furzer et al. 2021; Lopes-Rosa et al. 2017). This pattern imposes significant economic and social burdens, with relapses after abstinence harming their livelihoods (Erskine et al. 2014). More research is needed to develop effective substance use prevention programmes for the youth who are challenged by relapses, particularly in Lobatse, Botswana.

Programmes designed for youth substance users (YSUs) play a crucial role in helping them manage impulses, mitigate cravings and prevent relapse, while also fostering social skills and promoting the ability to defer gratification (WHO-UNODC 2020). Despite the elevated relapse rates among the youth, research on relapse prevention and recovery has only recently started to gain traction, especially in developed countries. While SUR poses a significant challenge for many nations worldwide, prevention efforts have received less attention, particularly in Africa; Lobatse in Botswana is not an exception. In Western countries, effective SUR prevention programmes have successfully reduced relapses; however, similar initiatives remain scarce and under-researched in Africa. A substantial body of evidence suggests that effective prevention strategies must take into account the social, cultural, environmental and individual factors that affect those with substance use issues. Therefore, this study aimed to develop a SUR prevention programme tailored to the local context (Appiah et al. 2018; Kabisa et al. 2021; Maikano et al. 2021).

There is no documented programme for preventing SUR in Lobatse, Botswana, and this study fills this epistemological and practice-based gap. To achieve this for Botswana, the researcher developed and validated a SUR prevention programme by synthesising the existing perspectives in the literature regarding programmes for preventing SUR and incorporating them with perceptions from the YSUs and psychiatric mental health nurses (PMHNs).

### Problem statement

Substance use relapse is a prevalent public health issue that jeopardises the lives of young individuals and has a considerable negative effect on their families and communities (Oladele 2021). Studies on youth worldwide clearly identify risk factors for substance use (Arora et al. 2015; Merikangas & McClair 2012). Peer pressure, substance availability and media portrayals of substance use as enjoyable impact young people’s initiation, continuation and relapse into substance use (Feinstein et al. 2012; Okafor 2020).

Youth substance users frequently undergo cycles of relapse, alternating between periods of active substance use and intervals of treatment and abstinence, sometimes spanning many years (Strang et al. 2020). The cost of treatment during these relapses can be significant, yet it tends to offer limited long-term benefits. Research indicates that approximately 65% – 70% of YSUs return to using their primary substances within 3 months following treatment, and two-thirds relapse within a year (Gonzales-Castaneda et al. 2022). Consequently, preventing relapse among the youth has become a crucial public health objective on a global scale. In Botswana, YSUs often experience SUR because of peer pressure, inadequate family support, easy access to substances in the community and a lack of follow-up care after discharge.

Research on SUR prevention has not been conducted in Botswana, raising concerns, particularly given that some sub-Saharan African countries report SUR rates as high as 59.9% to 75% (Appiah 2022; Kabisa et al. 2021; Swanepoel, Geyer & Crafford 2016). However, expert anecdotal notes have highlighted SUR as a significant concern in Botswana, along with a lack of prevention strategies (Gaolaolwe et al. 2025). The significant prevalence of SUR among youth creates a challenging cycle that must be addressed. This study proposes a prevention programme designed to support YSUs in achieving long-term sobriety and healthy, substance-free lives.

## Research methods and design

The study followed a multiphase approach in which an integrative literature review (ILR) and qualitative, descriptive, exploratory and contextual studies were conducted. The study sought to develop and validate a programme aimed at preventing SUR among youth and was conducted in three phases: the literature review phase, the empirical phase and the development and validation phase. During the literature review phase, an ILR was conducted (Gaolaolwe et al. 2025) in accordance with the guidelines set forth by Whittemore and Knafl (2005). This process adhered to the step-by-step guide for conducting an ILR, as outlined by Toronto and Remington (eds. 2020), which includes problem identification, literature search, data evaluation, data synthesis and analysis. The identified problem for this ILR stemmed from study reports and anecdotal notes from experts, which highlighted SUR as a significant concern in both Botswana and globally, because of a lack of prevention strategies. During the literature search, all relevant literature referring to a SUR prevention programme for youth with substance use problems was reviewed. This meant excluding studies that did not focus on SUR prevention programmes. For data evaluation, the ILR underwent a critical appraisal for quality using the 2018 Mixed Methods Appraisal Tool (MMAT). Consequently, commentaries, letters and editorials were excluded, as the review focused on theoretical, empirical and expert reports on the topic. The literature utilised encompassed diverse methodologies, including experimental and non-experimental studies, which offered various perspectives on the prevention of SUR among youths. However, the review incorporated grey literature that provided relevant information in addressing the research question. The data were extracted and synthesised using content analysis (Lubbe, Ten Ham-Baloyi & Smit 2020). The review was conducted to answer the following research question:

‘What does the published literature say about a substance use relapse prevention programme for young people?’

In the empirical phase, two qualitative exploratory descriptive studies were conducted on two distinct populations: PMHNs (step 1) and YSUs (step 2), all recruited from a psychiatric hospital in Lobatse, Botswana. The studies were conducted concurrently. The former examined the perceptions of PMHNs regarding SUR prevention in Lobatse, and the latter also focused on the perceptions of YSUs on how SUR can be prevented among the youth. Purposive sampling was used to select study participants for the empirical phase, with a sample size of 15 participants for each of the two strands. Semi-structured interviews were conducted with both populations, guided by a series of semi-structured questions. The interviews took place at a psychiatric referral hospital in Lobatse. The interviews enabled the collection of critical contextual data, including field notes, which are essential for qualitative research (Farooq & De Villiers 2017). The data from both the PMHNs and the YSUs were collected simultaneously, with equal emphasis and priority given to both groups. The researchers maintained rigorous standards throughout the data collection process by being open and transparent. Data analysis was conducted separately by the researchers and a co-coder using the thematic analysis put forth by Braun and Clarke (2012). Key concepts were identified and categorised using the themes and subthemes produced by the ILR and qualitative research. Data from different sources were combined and arranged using the six components of the survey list that Dickoff, James and Wiedenbach (1968) suggested in order to accomplish the classification. The programme was then developed based on insights from the ILR and the qualitative studies, adhering to the practice-oriented theory (POT) established by Dickoff et al. (1968) and the TALER protocol (Scott & Dennis 2011).

According to Dickoff et al. (1968), the six key elements are agent, recipient, context, process, dynamics and terminus. TALER stands for tracking, assessment, linking, engagement and retention. These elements systematically address essential components of an individual’s recovery journey, thereby decreasing the likelihood of relapses (Scott & Dennis 2011). The TALER protocol, a structured recovery management care approach, was integrated into six elements by Dickoff et al. (1968) to create synergy and improve engagement among YSUs in treatment. This integration aimed to enhance the programme outcomes and reduce relapse rates. Following the classification of concepts, a visual conceptual framework for the prevention of SUR among youth in Lobatse was developed, which served as a guide for creating the SUR prevention programme.

### Trustworthiness

Proper scientific methods were followed in conducting the ILR to ensure the validity of each study’s results and avoid compromising them. In the empirical phase, trustworthiness was ensured through the credibility and dependability of the study, as well as its transferability and confirmability, as per Lincoln and Guba’s framework (Polit & Beck 2017). The study’s credibility relied on confidence in the data derived from the informants’ lived and perceived experiences (Grove & Gray 2022; Polit & Beck 2017). To ensure confirmability, the researcher created an audit trail by collecting all relevant documentation for the study, including interview transcripts and field notes. This served as evidence of the methodological processes followed. To enhance the dependability of the study, the researcher provided a detailed description of the methods used for data collection, analysis and interpretation. To establish the transferability of the study, the researcher also included a comprehensive description of the study population, detailing the participants’ demographics and geographic boundaries.

### Ethical considerations

The study had ethical approval from the North-West University Health Institutional Review Board (Ethics number: NWU-00174-23-A1). Permission to conduct the study was obtained from the Ministry of Health, Botswana (Ref no: HPRD:6/14/1) and Sbrana Psychiatric Hospital in Lobatse (Ref: 4/2/2 III). The ethical principles of justice and autonomy were observed throughout the study. The researcher and supervisors of the study were university lecturers and had no undue influence on the participants.

## Results

The ILR identified three main themes regarding what is published on SUR prevention programmes for youth: continuing care, technology-mediated recovery management interventions and relapse prevention through developmentally engaging activities (Gaolaolwe et al. 2025). The subthemes of continuing care included post-treatment check-ups, tracking and assessment, engagement and retention and the utilisation of recovery support services. The subthemes related to technology-mediated recovery management were mobile aftercare interventions and internet-based relapse prevention strategies. In terms of relapse prevention and recovery promotion through engaging activities, the subthemes included physical activities, self-monitoring and educational initiatives that enhance literacy on substance use (Gaolaolwe et al. 2025).

Building on these insights, the empirical phase was conducted to explore the perceptions of PMHNs and YSUs regarding the prevention of SUR among the youth with substance use problems. The results of the empirical phase are divided into two steps. The first step presents the findings from a qualitative study conducted to explore the perceptions of PMHNs regarding the prevention of SUR among the youth with substance use problems. This step yielded two main themes: perceptions of PMHNs on the causes of youth SUR and perceptions of PMHNs on the prevention of youth SUR. The following subthemes emerged regarding the perceptions of PMHNs on the causes of SUR: psychological triggers of SUR, family causes of SUR and societal causes of SUR. Three subthemes emerged from the theme regarding the perceptions of PMHNs on the prevention of youth SUR as follows: individual interventions to prevent SUR, social and community interventions to prevent SUR, and healthcare interventions to prevent SUR.

The second step of the empirical phase explored the perceptions of YSUs on the prevention of SUR, and it also yielded two themes: perceptions of YSUs on the causes of SUR and perceptions of YSUs on the prevention of SUR. The following were the three subthemes for the perceptions of YSUs on the causes of SUR: psychological causes of SUR, social causes of SUR and healthcare barriers to prevent SUR. Four subthemes emerged on the perceptions of YSUs on the prevention of SUR as follows: individual interventions to prevent SUR, social interventions to prevent SUR (social support), community interventions to prevent SUR (community awareness and education), and healthcare interventions to prevent SUR. In the third phase (development phase), the results from the first two phases of the study were combined in a conceptual framework (i.e. classification of concepts) to develop the proposed SUR prevention programme, guided by the POT by Dickoff et al. (1968) and the TALER protocol. Concepts were classified through synthesis and integration of the results from the two phases (ILR phase and empirical phase). The developed conceptual framework was ultimately used as a guide and structure for developing the programme as shown in the discussion section of this study.

## Discussion

**FIGURE 1:**
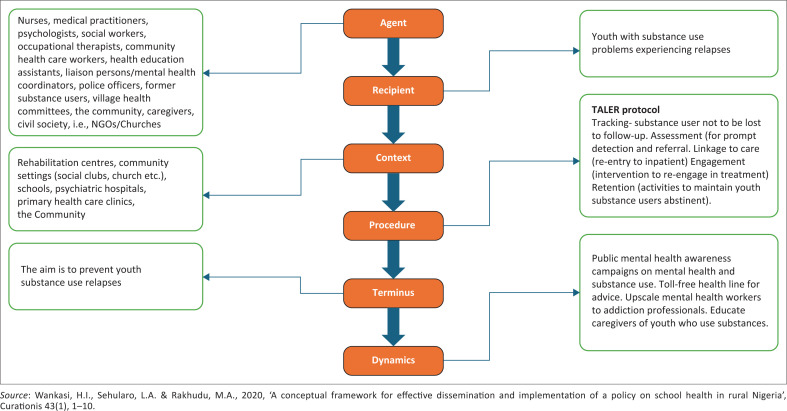
Concept classification, utilising the steps outlined in Dickoff et al. (1968).

The researchers engaged a creative process in which interrelated ideas from the results of the ILR and empirical phase (two qualitative exploratory, descriptive, contextual studies) were synthesised and integrated to develop a programme, guided by the POT by Dickoff et al. (1968) and the TALER protocol (Scott & Dennis 2011). Firstly, the researchers generated themes and subthemes from the ILR and the qualitative studies. Secondly, the researchers identified and classified concepts from the ILR and qualitative studies, and clustered them according to the six survey list elements proposed by Dickoff et al. (1968). Thus, the data from these different sources were consolidated to design a SUR prevention programme. According to Dickoff et al. (1968), the six elements are agent, recipient, context, process, dynamics and terminus. To foster synergy and enhance outcomes, the TALER protocol was incorporated – a structured approach to managing substance use recovery – into the strategy for preventing SUR. The TALER protocol (tracking, assessment, linking, engagement and retention) methodically addresses crucial elements of substance use recovery (Scott & Dennis 2011). This protocol provided valuable insights for the fifth component of Dickoff’s process, serving as a roadmap for the implementation of the programme. Following the classification of concepts, a visual conceptual framework (Figure 1) for the prevention of SUR among youth in Lobatse was presented, which then guided the development of the SUR prevention programme as discussed in the subsequent subheadings.

### Purpose of the programme (terminus)

The terminus answers the question, ‘What is the end product of the programme?’ (Dickoff et al. 1968). The purpose of the programme is to prevent SUR among the youth in Lobatse, Botswana. The programme agents – psychiatric nurses, social workers, medical officers and psychologists – are expected to prevent SUR among the youth with substance use problems who experience relapses after a period of abstinence.

### Agents of the programme (Who is the agent of the programme?)

The agent for this programme is a person who is responsible for executing the programme’s activities geared towards preventing SUR (Dickoff et al. 1968). For effective SUR prevention, key stakeholders must collaborate inter-disciplinarily and inter-sectorally as agents for this programme. The stakeholders should include the multidisciplinary team (MDT) such as medical practitioners, occupational therapists, nurses, police and youth who successfully recovered from substance use, government and civil society such as churches and non-governmental organisations (NGOs) offering SUD rehabilitation, community members and families or caregivers of YSUs.

The medical practitioners, for example, the psychiatrists and medical officers, as agents of this programme, should allow for ease of re-entry (re-admission) of the YSUs into treatment without complicated admission procedures if they report being overwhelmed and on the verge of a relapse. The ease in admission processes of YSUs is expected to encourage them to seek treatment or health services promptly. The social workers should work collaboratively with PMHNs in conducting family support visits after the discharge of YSUs from inpatient care as part of aftercare and offer support. Psychologists should provide counselling and psychotherapy to YSUs, from inpatient to outpatient, to equip them with the resources, insights and support needed to navigate the challenges of their recovery journey. The psychologists should build the YSUs’ resilience and equip them with strategies that they can employ to reduce the risk of relapse. The YSUs are also themselves part of the agents of this programme, and they are expected to willingly participate in their recovery for this programme to succeed.

In each district, a PMHN or a nurse trained in addiction must be appointed as a liaison manager, a linkage person or a district health management team (DHMT) mental health coordinator who shall coordinate substance use prevention activities in their catchment area as agents of this programme. This programme uses the title, ‘DHMT mental health coordinator’, which is synonymous with the liaison manager or linkage person to suit the operations of the local context. Upon discharge of the YSUs from inpatient treatment, the discharging institution must notify the DHMT mental health coordinator, who should, in turn, inform the nurses in the primary health care (PHC) setting about this discharge. Then, the nurses and other members of the MDT (social workers, health education assistants, etc.) should schedule and conduct home visits as part of follow-up or continuing care for the discharged YSU. During home visits, the nurses (especially PMHNs) or the healthcare providers who are agents of this programme (especially those trained as addiction professionals) should assess how the YSU keeps up with the discharge plan and how the family copes and provides support and psychoeducation.

The agents of this programme, such as the healthcare providers, should work collaboratively with the available community health structures. Thus, community-based health structures such as the village health committees (VHCs) should be initiated or revived to collaboratively work with nurses and the MDT in the follow-up of YSUs in the community and in initiatives and activities aimed at prevention, treatment and rehabilitation. The VHC plays a significant role as agents of this programme in developing and linking the network of religious, indigenous and professional services and building relationships that support the long-term recovery of YSUs and their families. The VHC is expected to participate in mental health awareness and education campaigns, thus fostering community support and understanding, all of which remove barriers to recovery (e.g. stigma) and enhance individuals’ recovery capital. Civil societies such as the church and NGOs should also be involved as part of a greater community. For instance, upon discharge from the inpatient setting, the mental health coordinator can inform NGOs about the discharge for follow-up care. Non-governmental organisations can also house recovery coaches who can critically help in relapse prevention for those in recovery and offer valuable SUR prevention services such as counselling, psychotherapy, detox programmes and outpatient rehabilitation services as part of the community recovery capital.

Lawmakers should enact stringent laws that target drug dealers to curtail the sale of substances and thus limit their availability in the community. Thus, the law should continue to criminalise the sale of illicit substances, a move that is expected to reduce their availability, which is associated with increased use and relapses. The police should conduct searches at the border posts and shebeens to curb the trafficking of drugs into the communities, which is a contributing factor to SUR. The law enforcement efforts, such as raids, and other operations, are expected to have a deterrent effect on the production, supply and distribution networks of illicit drugs in communities.

The families of the YSUs should receive counselling and psychoeducation on SUD as a mental illness to scaffold their understanding and support in the recovery process of their YSUs who experience SUR. An understanding family, as an agent of this programme, is expected to avoid the use of stigmatising words towards their family member with substance use problems. The family should support the YSU’s recovery journey by actively participating in their care, for example, monitoring for signs of relapse and triggers and intervening appropriately as guided through family counselling and psychoeducation.

The YSUs who have recovered from substance use and can withstand relapses should be used as agents of the programme for peer-led education in which they can share insights into their recovery trajectory. It is expected that learning from the survivors of SUR would help YSUs appreciate the challenges posed by triggers to relapse and strategies that can be employed to overcome them. As agents of this programme, the YSUs who have since recovered from substance use are expected to provide a valuable account of the real-life experiences of SUR that may either be overlooked or not be amenable to the theoretical approaches that the health workers use.

The community should support the YSUs from relapsing by creating an environment that is supportive of recovery. Thus, PMHNs and the MDT should run public mental health campaigns and education to raise community awareness about substance use to foster their understanding and reduce stigma towards substance users. As an agent of this programme, an informed community is expected to actively and proactively offer support through the provision of employment and volunteer opportunities to keep the YSUs engaged and prevent their idleness and boredom, thus preventing SUR. Institutions of higher learning where most of the programme targets are should be included as stakeholders wherein student welfare officers, student counsellors and university or college nurses are involved in students’ follow-up in colleges or universities.

### Recipients of this programme (Who is the recipient of the programme?)

The recipients of the programme are youth aged 18 to 24 years old who experience SUR when they have quit and want to abstain. When the youth participate in this programme, they develop increased awareness and understanding of the triggers of their relapses and employ the learnt coping strategies to prevent a relapse.

### Context of the programme (In which context will the programme be implemented?)

The programme can be offered in different contexts such as families, communities, community rehabilitation centres, NGOs and health facilities such as psychiatric hospitals and PHC clinics.

### Procedure of the programme (What is the guiding protocol of the programme?)

This part answers the question, ‘What are the guiding procedures or protocols or techniques to implement this programme?’ (Dickoff et al. 1968). The guiding procedure for the programme is the TALER protocol. The protocol facilitates the management of addiction and relapses through recovery management check-ups (RMCs). By espousing the TALER as the guiding protocol for the programme, the check-ups and home visits should not be scheduled to take long to curtail the recurring and cyclical nature of substance use via monitoring and linkage to treatment.

### Tracking

Tracking entails monitoring the substance user’s recovery progress (ISSUP 2020; Scott & Dennis 2011). This approach identifies triggers and behavioural patterns that are potential risk factors for relapse. The YSUs should be tracked to ensure that they keep on course with treatment plans to prevent SUR. The YSUs not only relapse to substances but also miss medical checkups. Thus, the essence of tracking is to monitor YSUs continuously to keep them on the course of their recovery trajectory. The agents of the programme should proactively track the YSUs and not solely rely on them to honour medical appointments. Thus, stakeholders such as the health care service providers, NGOs, among others, especially those operating at the PHC level, should always endeavour to know if the YSU still resides in their catchment area of operation and inform the mental health coordinator of any changes. Home visits should be conducted as part of tracking the YSU to encourage them to honour medical check-ups and apply the skills learnt to resist cravings and avoid triggers. In addition, the YSUs should be empowered to take responsibility for their own monitoring, with family members being invited to also participate in their monitoring. The intensity of tracking should differ from one individual to another, with those with a high propensity to relapse tracked weekly or fortnightly. Additionally, tracking can be conducted through telephone and internet communication, in which a brief structured assessment of risk for relapse is conducted for further intervention.

### Assessment

Assessment of the YSUs should be conducted to ensure that they are on track with the gains made from inpatient treatment (Scott & Dennis 2011). Different members of the MDT can conduct various assessments. These include, but are not limited to, behavioural and coping skills assessments, evaluations of support systems, identification of stressors and triggers, functional assessments, substance use history assessments and relapse risk assessments. Performing these assessments regularly throughout the tracking process assists YSUs clearly understand their recovery journey and prevent SURs. This practice enables timely interventions and adjustments to treatment plans, fosters open communication between individuals and their support networks and ultimately enhances the likelihood of successful long-term recovery.

Assessment should be a continuing activity from medical consultation during medical reviews at outpatient departments to YSUs’ places of abode during home visits. The assessment conducted during home visits should target both the YSU and their families to identify the triggers to relapse and offer support such as counselling, psychoeducation or referral to other stakeholders for further intervention. The family should provide a report of their assessment of how the YSU progresses at home. The YSU should also provide a self-report on their progress and the challenges encountered in maintaining abstinence to aid prompt action. As for the outpatient assessments, they should be conducted at least quarterly for about 4 years following the discharge of the YSU from inpatient rehabilitation. The YSU should give a self-report on triggers and abstinence status, which should be corroborated by an accompanying family member and verified by urine tests.

### Linkage

Linkage refers to the process of connecting individuals who are in recovery to vital resources and support systems that aid in maintaining abstinence and preventing relapse (Karno et al. 2021; NIDA 2018; Scott & Dennis 2011). It involves establishing and cultivating positive relationships with various services that address SUDs, including NGOs, support groups and networks that are essential to the recovery journey. Effective linkages to resources for SUR prevention typically necessitate a collaborative effort among multiple stakeholders. The agents of this programme should unite into a comprehensive support network for those in recovery. Each player contributes by identifying needs, facilitating access to services and enhancing the overall support system. This teamwork ultimately helps to diminish the risk of relapse and fosters long-term recovery.

In cases where a YSU in the community is assessed and a relapse is imminent, suspected or real, the coordinator or the MDT should easily link them back to inpatient treatment, residential services, SUD outpatient services or community support services to prevent a full relapse or minimise its impact.

### Engagement

In the substance use recovery process, engagement involves the active participation of individuals with substance use issues in their healing process, as well as the creation of meaningful connections with available services, support systems and resources (Scott & Dennis 2011). The MDT should play a crucial role in promoting engagement in substance use treatment and recovery by providing personalised, integrated care and adopting a collaborative approach. By creating a supportive environment, MDTs enhance the commitment of YSUs to their recovery journey. This collaboration to achieve engagement not only addresses the complex nature of addiction and relapses but also empowers YSUs to take an active role in their own treatment and recovery. Thus, problems such as roadblocks found throughout the admission process should be identified and avoided to encourage the YSUs to more easily ‘show’ up for treatment following referral. Also, the agents of this programme should notify the mental health coordinator as a linkage person in cases where the YSU makes threats to stop treatment or misses an outpatient visit, and the coordinator should set up an intervention to encourage re-engagement in treatment.

### Retention

Retention is the sustained involvement of YSUs facing substance use challenges in their treatment programmes once they embark on the journey of recovery (Scott & Dennis 2011). When the YSUs remain engaged in their treatment, it reflects a deep commitment to their recovery plan. Efforts by the agents of this programme to attain YSUs’ ongoing participation in treatment are vital, as it would not only enhance the likelihood of achieving positive therapeutic outcomes but also realise retention and significantly reduce their chances of relapse. In essence, strong retention rates serve as a crucial indicator of the YSUs’ dedication to overcoming their substance use issues.

Occupational therapy (OT) should aim to assist YSUs in achieving independence and enhancing their quality of life through meaningful activities. These activities are crucial for retention and successful reintegration of YSUs into their daily lives and communities. Occupational therapy should offer interventions that help restore previous roles and promote a healthier lifestyle. Occupational therapists should support YSUs by utilising goal-directed activities tailored to their individual needs. This may include fostering emotional insight into addictive behaviours, facilitating their return to work, providing supportive aftercare, encouraging the exploration of new positive activities and developing social skills. Adequate resources such as human capital (occupational therapists, psychologists, addiction professionals, etc.) and physical resources (e.g. rehabilitation centres) are required to achieve adequate (many) sessions for inpatient and outpatient therapy to achieve retention. Allowing YSUs to stay in therapy or treatment for 3 months or more is necessary and can reduce the chances of relapse and aid in recovery.

Appropriate pharmaceutical management for those experiencing cravings and withdrawal symptoms is important to prevent relapses and sustain retention to treatment (Mitchell et al. 2021). The use of appropriate medication is expected to play a critical role in reducing cravings and withdrawal symptoms during detoxification, enhancing the likelihood that YSUs will remain engaged in treatment. Psychiatrists and pharmacists should recommend medication suitable for the local context, such as naltrexone for alcohol dependency, to effectively diminish cravings as support for continued participation in treatment. When withdrawal symptoms are effectively managed, YSUs are more inclined to benefit from other therapeutic interventions like cognitive-behavioural therapy (CBT). The comprehensive approach suggested by this programme underscores the importance of integrating medication into a holistic strategy to aid retention and prevent SUR.

### Dynamics (What are the dynamics of the programme?)

The dynamics refer not only to power bases like the agents, recipients and context but also to activities performed to ensure the success of the programme (Dickoff et al. 1968). The programme should be delivered physically. Additionally, it can also be offered virtually via technology-mediated educational activities that promote literacy on SUR as part of either mobile aftercare interventions or internet-based relapse prevention strategies (Beck et al. 2021). Successful implementation of the programme requires collaboration among all stakeholders. To ensure effective collaboration, the DHMT mental health coordinators should oversee all community mental health activities related to this programme within their districts. The coordinator should also conduct workshops and in-service education sessions to engage stakeholders who are agents of this programme, helping them understand the programme and fostering acceptance and support.

To prevent relapses caused by a shortage of substance use rehabilitation facilities, the Ministry of Health should establish rehabilitation centres and ensure they are adequately staffed with qualified mental health and addiction professionals. These centres should specialise in substance use rehabilitation and offer personalised interventions on an inpatient and outpatient basis. It is expected that with the availability of these rehabilitation centres, individuals can receive the extended support they need to facilitate their recovery and reduce the likelihood of relapses. In addition, there is a need for some community-based, youth-friendly halfway homes and facilities that offer outpatient care to promote the uptake of SUD management services by the YSUs. These facilities are expected to provide vital services that encourage recovery, support healthy development and foster a sense of community. By ensuring a safe environment and employing an evidence-based approach, they can deliver tailored care that effectively helps prevent SURs among the youth.

Community recreational and vocational facilities should be established and tailored for YSUs in outpatient treatment for substance use to enhance their recovery experience and prevent their relapses. Community recreational and vocational facilities are expected to foster supportive environments that promote well-being, social integration and life skills. To aid recovery through recreation, even rehabilitation centres should develop scheduled recreational activities as part of the treatment plan, which should include fitness classes, sports, arts and crafts, among others. The recreational activities should be aligned with the therapeutic goals of YSUs to assist them in developing positive social skills, stress management and coping skills. Rehabilitation centres should have structured, inclusive and enjoyable recreational activities within the rehabilitation programmes to build a sustainable, healthy lifestyle post-treatment.

The health workers who are agents of this programme should work collaboratively to ensure its success. Diverse therapists, such as psychotherapists, occupational therapists, among others, should ensure that YSUs undergo intense therapy before they are discharged from inpatient care to equip them with the necessary skills and strategies to cope with stress and resist triggers. To maintain the gains achieved from inpatient rehabilitation, the PMHNs, social workers and health care assistants should continue care by conducting home visits and outreaches to ensure that the YSUs and their families stay focused on the treatment plan. Virtual check-ups should also be used to augment the physical reviews and home visits.

Another gap in dealing with SUDs is the shortage of addiction professionals. Efforts should be made to upskill mental health professionals and MDT by training them as addiction professionals. Also, the NGOs should be assisted to professionalise and be accredited as well. Even the individuals who are recovery coaches should undergo an accredited programme to guard against harming YSUs. Also, some health workers in PHC should receive additional training as addiction professionals. The trained community-based addiction personnel could lead the community initiatives aimed at combating substance use issues within their localities. Their presence can facilitate access to culturally competent care and support, helping to address gaps in services and meet the comprehensive needs of individuals struggling with SUDs and SURs. By focusing on prevention, early intervention, treatment and recovery support, community-based addiction personnel can contribute to building healthier communities and reducing the chances of SUR.

### Validation of the programme

Experts in SUDs validated the programme through an e-Delphi process. This process was conducted over three successive rounds until consensus was achieved. The experts were asked to review a comprehensive draft programme for preventing SUR and evaluate it using a Likert scale, with the option to provide written comments to express their opinions. Ratings from the e-Delphi participants were collected across the three rounds, and comparisons were made among the responses of participating experts after each round. Additionally, suggestions for improving the programme were documented from all rounds. Throughout the e-Delphi process, the participants remained anonymous to each other, with the researcher facilitating controlled feedback (Barkhordari Ahmadi et al. 2023). A 100% consensus was finally reached in the third round.

### Recommendations for future research

Studies employing more rigorous designs, such as randomised control trials, can be conducted to evaluate the effectiveness of this programme.

### Implications for practice

To the best of our knowledge, this is the first study conducted on the development of a SUR prevention programme in Lobatse. The programme presents a comprehensive strategy for preventing SUR, offering actionable approaches for nurses and mental health professionals to address the unique needs, triggers and circumstances of YSUs in Lobatse. It underscores the vital role that nurses and the healthcare team play in crafting aftercare plans that prioritise ongoing support and sustained engagement in recovery activities for youth experiencing SUR challenges. This programme could be adapted and implemented by nurses and mental health care workers in similar settings or environments.

### Limitations of the study

The programme was developed based on the findings of one town in Botswana, that is, Lobatse, and cannot be generalised to YSUs in other contexts. However, the programme could be extrapolated or applied in other similar settings.

## Conclusion

Substance use relapse is a daunting and multifaceted challenge that requires a robust, dynamic and comprehensive intervention programme to address its causes and prevent recurrence. This programme addresses the gap that exists in the prevention of SUR in Botswana. The programme was developed based on the results of an ILR in the study’s first phase and some qualitative research in the empirical phase. The study findings from these phases confirm what the programme should entail and provide impetus for the aftercare of the YSUs to prevent their relapses (i.e. their continuous assessment and the creation of a supportive environment). This unique programme, which is the first of its type in the country, contributes important knowledge to the field of mental health and psychiatry and is expected to enhance the quality of care for the youth experiencing SURs after a period of abstinence.
